# Protective Effect of Two Alkaloids from *Hippophae rhamnoides* Linn. against Doxorubicin-Induced Toxicity in H9c2 Cardiomyoblasts

**DOI:** 10.3390/molecules26071946

**Published:** 2021-03-30

**Authors:** Wenna Zhou, Jian Ouyang, Na Hu, Gang Li, Honglun Wang

**Affiliations:** 1 Department of Life Sciences and Health, QiuZhen College, Huzhou University, Huzhou 313000, China; 02638@zjhu.edu.cn; 2 CAS Key Laboratory of Tibetan Medicine Research, Northwest Institute of Plateau Biology, Xining 810008, China; ygzjj@126.com (J.O.); huna@nwipb.cas.cn (N.H.); 3 Center for Mitochondria and Healthy Aging, College of Life Sciences, Yantai University, Yantai 264005, China; 4 Huzhou Plateau Biological Resource Centre of Innovation, Northwest Institute of Plateau Biology, Chinese Academy of Sciences, Huzhou 313000, China

**Keywords:** alkaloid, doxorubicin, apoptosis, mitochondrial function, seabuckthorn

## Abstract

Background: Doxorubicin (Dox) is one of the most frequently prescribed anti-cancer drugs. However, clinical application with Dox is limited due to its potentially fatal cumulative cardiotoxicity. N-*p*-coumaroyl-4-aminobutan-1-ol (alk-A), an organic amide alkaloid and hippophamide (alk-B), a rare pyridoindole alkaloid were successfully obtained by purification and separation of seabuckthorn seed residue in our previous research. This study was undertaken to investigate the protective effect of alk-A and alk-B against Dox-induced embryonic rat cardiac cells (H9c2 cells) apoptosis. Methods: H9c2 cells were treated with Dox (2.5 µM) in the presence of alk-A and alk-B (10, 20, and 40 µM) and incubated for 24 h. Results: It was shown that pretreatment of the H9c2 cells with alk-A and alk-B significantly reduced Dox-induced apoptosis. Alk-A and alk-B both inhibited reactive oxygen species (ROS) production and suppressed cleaved-caspase-3 protein expression and the activation of JNK (Jun N-terminal kinases), as well as increasing ATP levels, favoring mitochondrial mitofusin protein expression, and relieving damage to mitochondrial DNA. Conclusions: These results suggest that alk-A and alk-B can inhibit Dox-induced apoptosis in H9C2 cardiac muscle cells via inhibition of cell apoptosis and improvement of mitochondrial function, while alk-B showed more protection. Alk-B could be a potential candidate agent for protecting against cardiotoxicity in Dox-exposed patients.

## 1. Introduction

Doxorubicin (Dox), an anthracycline analog, and is one of the most frequently prescribed anti-cancer drugs. It is widely used in the treatment of several cancers such as leukemia, lymphoma, breast, lung, and other solid tumors [1]. As with other anticancer agents, its clinical use is limited due to detrimental side-effects such as cumulative, dose-related, progressive myocardial damage that may lead to congestive heart failure (CHF) [2]. Research has indicated that an estimated cumulative 26% of patients would experience doxorubicin-related CHF at a cumulative dose of 550 mg/m^2^ [3]. The intracellular targets and the molecular mechanisms involved in Dox-induced cardiotoxicity are not completely understood, but previous studies have contributed to the theory that this is, in fact, a multifactorial process that leads to cardiomyocyte death as the terminal downstream event [4,5,6,7,8]. 

The abundance of mitochondria in cardiomyocytes closely links mitochondrial function with myocardial function. Mitochondrial dysfunction has become an apparent hallmark of Dox-induced cardiotoxicity and during the last decade, evidence has accumulated to support the critical role of mitochondria in determining the fate of cardiomyocytes [4]. One of mechanisms implicates mitochondria in the key roles of the intrinsic apoptotic pathway during Dox-induced cardiotoxicity—disruption of the ETC (electron transporting respiratory chain) and ATP production, release of proteins that trigger the activation of the caspase family of proteases, and alterations in redox potential [9,10,11]. This cardiotoxicity emphasizes the urgent need for novel adjuvant therapeutic agents to increase clinical applications. Mitochondrial function can be used as an indicator of myocardial toxicity in drug therapy.

**Figure 1:**
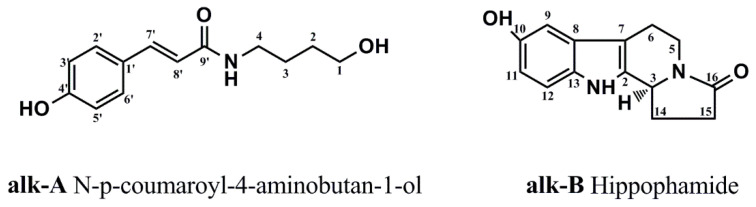
Chemical structures of alkaloids from *Hippophae rhamnoides* L.

The genus *Hippophae rhamnoides* Linn. subsp. sinensis Rousi (*Hippophae rhamnoides* L.), a plant of the family Elaeagnaceae, is an edible medicinal plant which has a long history in the treatment of ischemic heart disease and circulatory disorders in Tibetan medicine and traditional Chinese medicine [12,13]. Recently, several pharmacological studies have reported that chemicals existing in the seabuckthorn have shown a protective effect against drug-induced cardiotoxicity [12,14,15,16]. Abudureyimu Gulimire et al. reported that total flavonoids of seabuckthorn can increase myocardial GSH-Px (glutathione peroxidase) and SOD (superoxide dismutase) activities and decrease MDA (malondialdehyde) content to protect myocardial function against Dox-induced cardiotoxicity [17]. In view of the above-mentioned, the general approach to attenuate Dox-induced cardiotoxicity is to utilize antioxidants from seabuckthorn. Although antioxidants show promising results in in vitro tests, it is apparent that protection against Dox-induced cardiotoxicity in animal models seldom yields the same response in humans [18]. In recent years, accumulating evidences has demonstrated a variety of alkaloids that have attenuated Dox-induced cardiotoxicity in the embryonic rat cardiac cell (H9c2 cell) by increasing anti-oxidant and antiapoptotic ability and protecting mitochondrial function in vitro and in vivo [19,20,21,22,23]. Changying Hu et al. reported that total alkaloids from seabuckthorn can promote the survival rate of cardiomyocytes subjected to I/R (ischemic/reperfusion)injury and reduced the release of myocardial enzymes induced by I/R injury [14]. Interestingly, two new alkaloids were successfully separated and purified from seabuckthorn seed residue by our laboratory (chemical structures were shown in Figure 1) [24]. Therefore, our aim was to test the hypothesis that the alkaloid could prevent Dox-induced cardiomyocyte death and to identify the mechanisms involved in an in vitro model. 

## 2. Results

### 2.1. Alkaloids from Hippophae rhamnoides L. Decrease Dox-Induced Cytotoxicity in H9c2 Cells

**Figure 2:**
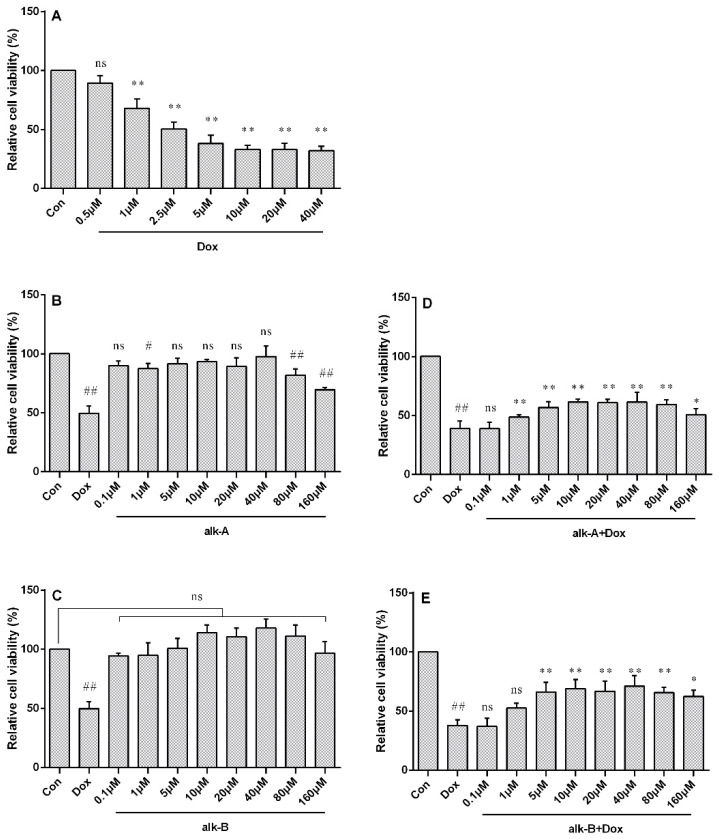
The effect of alkaloids on doxorubicin (Dox)-induced cytotoxicity in embryonic rat cardiac cells (H9c2 cells). Cell viability of H9c2 cells after treatment with different concentrations of Dox (**A**), cell viability of H9c2 cells after exposure to different concentrations of N-*p*-coumaroyl-4-aminobutan-1-ol (alk-A) (**B**), effect of alk-A on Dox-induced cytotoxicity in H9c2 cells; ^#^ *p* < 0.05 (**C**), cell viability of H9c2 cells after exposure to different concentrations of hippophamide (alk-B) (**D**), and effect of alk-B on Dox-induced cytotoxicity in H9c2 cells (**E**). ^##^ *p* < 0.01 vs. control, * *p* < 0.05, ** *p* < 0.01 vs. Dox treated group; **ns** means no significant difference.

In order to determine a treatment concentration of Dox, H9c2 cardiac cells were incubated with 0.5, 1, 2.5, 5, 10, 20, and 40 µM of Dox for 24 h and cell viability was assessed by MTT (3-(4,5-dimethylthiazol-2yl)-2,5 diphenyltetrazoliumbromide) assay. Dox inhibited cell proliferation in a dose-dependent manner as shown in Figure 2A. The half-maximal inhibitory concentration (IC50) of Dox toward the growth of H9c2 cells was determined to be ~2.5 µM, and this concentration was chosen for further experiments. As shown in Figure 2B,D, no significant toxic effect on cell viability was seen in the following treatment with alk-B for 24 h. While N-*p*-coumaroyl-4-aminobutan-1-ol (alk-A) showed a little cytotoxicity when its concentration was at 80 μM and 160 μM, cell viability was reduced to 81.74 ± 3.10% and 69.42 ± 1.21%, respectively. Interestingly, cells pretreated with alk-A and alk-B at different concentrations (0.1–160 μM) for 1 h, significantly decreased Dox-induced cytotoxicity in a dose-dependent mode as shown in Figure 2C,E. Alk-A and alk-B at 5, 10, 20, 40, and 80 µM significantly increased cell viability when compared to the Dox model group (*p* < 0.01). Concentrations of 10, 20, and 40 µM of alk-A and alk-B were chosen for this study, as they showed the most significant change in cell viability in comparison to the other treatment group.

### 2.2. Alkaloids Regulate Intracellular ROS Levels

**Figure 3:**
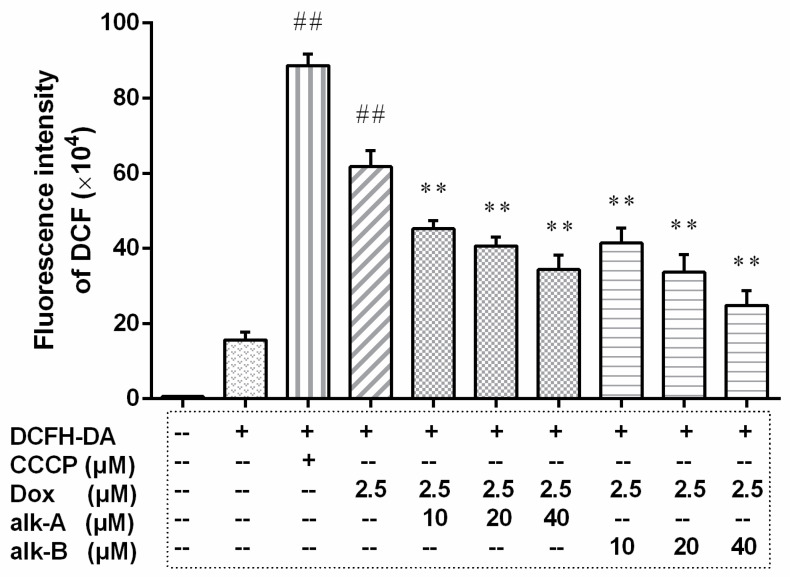
The effects of alkaloids on intracellular reactive oxygen species (ROS) level in 2.5 μM Dox-induced H9c2 cells. The cells were pretreated with different concentrations of alk-A and alk-B (10, 20, and 40 µM) for 1 h, then exposed to Dox for 24 h. CCCP (carbonyl cyanide 3-chlorophenylhydrazone) was used as a negative contrast group. Results are mean ± SEM from three independent experiments. DCFH-DA(2,7-Dichlorodi -hydrofluorescein diacetate) is a fluorescent probe to measure ROS level. ^##^ *p* < 0.01 vs. control, * *p* < 0.05, ** *p* < 0.01 vs. Dox group.

The effect of alkaloids against Dox-induced intracellular reactive oxygen species (ROS) was evaluated using the DCF (2′,7′-dichlorofluorescein) fluorescence method; the result is shown in Figure 3. Our data suggested that cells exposed to Dox exhibited a rapid increase of intracellular ROS and pre-treatment with alk-A and alk-B all resulted in a dramatic decrease of Dox-induced intracellular ROS in a dose-dependent manner. The addition of Dox (2.5 µM) to cardiac muscle cells caused a four-fold increase in the DCF fluorescence intensity (61.76 ± 2.50 × 10^4^) compared to the NC (Negative Control) group (15.68 ± 1.18 × 10^4^). Pre-treatment with alk-A of different concentrations (10, 20, and 40 µM) lowered the Dox-induced increase of DCF fluorescence to 45.20 ± 1.29 × 10^4^, 40.66 ± 1.38 × 10^4^, 34.39 ± 2.18 × 10^4^, respectively. Pre-treatment with alk-B at the same dose as alk-A (10, 20, and 40 µM) reduced the DCF fluorescence to 41.42 ± 2.30 × 10^4^, 33.65 ± 2.735 × 10^4^, 24.85 ± 2.24 × 10^4^, respectively. The result shown that the three dosages of alk-A or alk-B were all significantly different compared with the Dox group (61.76 ± 2.50 × 10^4^, *p* < 0.01). By comparison, the reduced effects of alk-B on intracellular ROS level was better than alk-A at the same dosage. 

### 2.3. Alkaloids Modulate Caspase-3 Protein Expression in Dox-Treated H9c2 Cells

**Figure 4:**
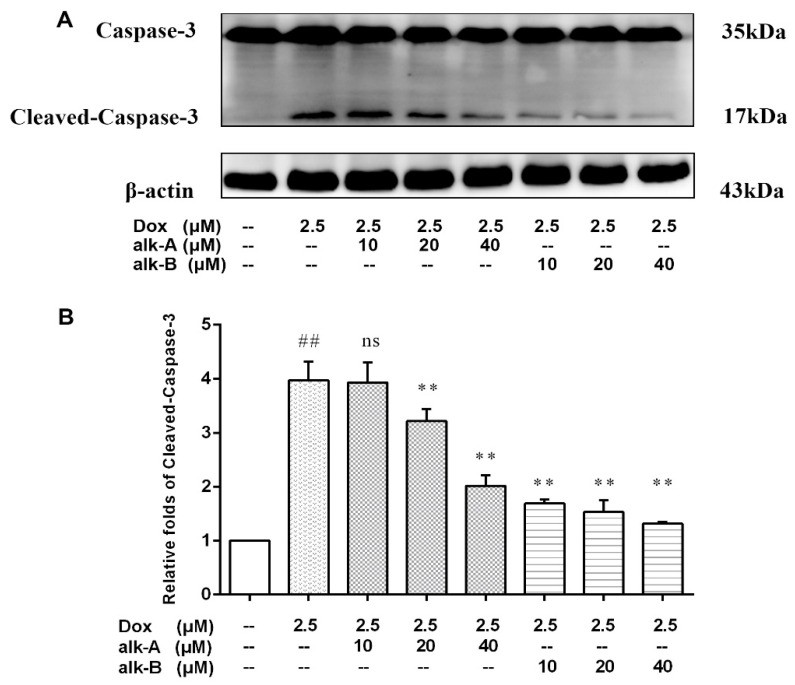
The effects of alkaloids on caspase-3 protein expression levels of Dox-treated H9c2 cells. The cells were pretreated with different concentrations of alk-A and alk-B (10, 20, and 40 µM) for 1 h, then exposed to Dox for 24 h (**A**). Relative folds of cleaved-caspase-3 (**B**). Results are mean ± SEM from three independent experiments. ^##^ *p* < 0.001 vs. control, * *p* < 0.05, ** *p* < 0.01 vs. Dox group.

In order to investigate the Dox-induced cell apoptosis, the expression level of cleaved-caspase-3 was examined (Figure 4). The relative expression level of cleaved-caspase-3 significantly increased (3.95-fold) after the addition of 2.5 µM Dox. Alk-A and alk-B dose-dependently inhibited activation of caspase-3, while 10 µM alk-A pre-treatment was ineffective in preventing the increase in caspase-3 activity. Both 20 µM and 40 µM alk-A pre-treatment limited the increase in caspase-3 activity to 3.22 ± 0.13-, and 2.02 ± 0.11-fold, respectively, over the normal control group value. Ten, 20, and 40 µM alk-B pre-treatment limited the increase in caspase-3 activity to 1.69 ± 0.041-, 1.53 ± 0.12- and 1.31 ± 0.23-fold, respectively, over the control value. The results showed that alk-B in prevented the increase in caspase-3 activation better than alk-A at the same dosage. 

### 2.4. Alkaloids Inhibit JNK Activation in Dox-Treated H9c2 Cells

**Figure 5:**
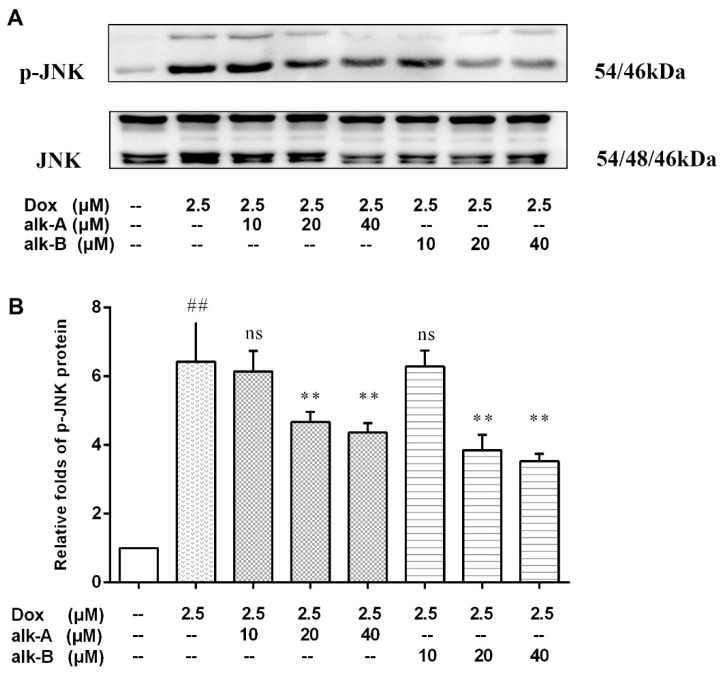
The effects of alkaloids on JNK-1/2/3 (Jun N-terminal kinases T183/T183/T221) protein expression level in Dox-treated H9c2 cells. The cells were pretreated with different concentrations of alk-A and alk-B (10, 20 and 40 µM) for 1 h, then exposed to Dox for 24 h (**A**). Relative folds of p-JNK (**B**). Results are mean ± SEM from three independent experiments. ^##^ *p* < 0.01 vs. control, * *p* < 0.05, ** *p* < 0.01 vs. Dox group.

To assess the cardio-protective effects of alk-A and alk-B on inhibiting Dox-induced apoptosis through the JNK-1/2/3 (Jun N-terminal kinases T183/T183/T221) of H9c2 cells we first measured p-JNK, p-ERK, and p-P38 expression by Western blotting (only the result of p-JNK is shown in this paper). H9c2 cells treated with 2.5 μM Dox rapidly induced the relative expression level of phosphorylation of JNK-1/2/3 (6.42 ± 0.68-fold vs. control group, *p* < 0.01). The degree of phosphorylation of JNK-1/2/3, compared to Dox group, was significantly suppressed by the administration of alk-A (4.67 ± 0.17-fold, and 4.37 ± 0.16-fold vs. control group) and alk-B (3.85 ± 0.26-fold and 3.54 ± 0.12-fold vs. control group) under 20 and 40 μM concentration conditions, as shown in Figure 5. However, 10 µM alk-A and alk-B pre-treatment was ineffective in preventing the increase in phosphorylation of JNK-1/2/3.

### 2.5. Alkaloids Inhibit Mitochondrial Dysfunction in Dox-Treated H9c2 Cells

Because mitochondrial dysfunction was accompanied by increased ROS levels in Dox-induced apoptotic cells, we further investigated the total cellular ATP production, mitochondrial fusion protein, and mtDNA damage to assess the effects of alkaloids on mitochondrial function.

#### 2.5.1. The Effect of Alkaloid Treatment on ATP-Relative Contents in Dox-Treated H9c2 Cells

**Figure 6:**
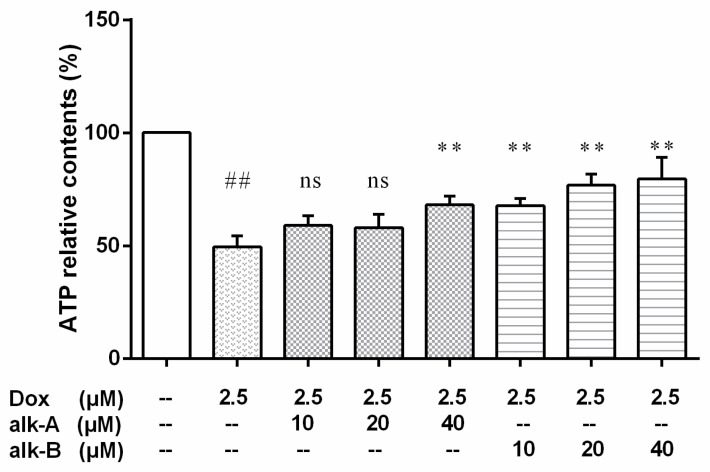
The effects of alkaloids on the ATPs level of Dox-treated H9c2 cells. The cells were pretreated with different concentrations of alk-A and alk-B (10, 20, and 40 µM) for 1 h, then exposed to Dox for 24 h. Results are mean ± SEM from three independent experiments. ^##^ *p* < 0.01 vs. control, * *p* < 0.05, ** *p* < 0.01 vs. Dox group.

Our data suggested that Dox treatment caused a decrease in intracellular ATP synthesis (Figure 6). Pre-treatment with 40 µM alk-A or 10, 20, or 40 μM alk-B could significantly raise the Dox-induced reduction of intracellular ATP contents. However, 10 μM or 20 μM dosage of alk-A was ineffective in raising the intracellular ATP levels. 

#### 2.5.2. Alkaloids Modulate the Expression Levels of Mitochondrial Fusion Proteins in Dox-Treated H9c2 Cells

**Figure 7:**
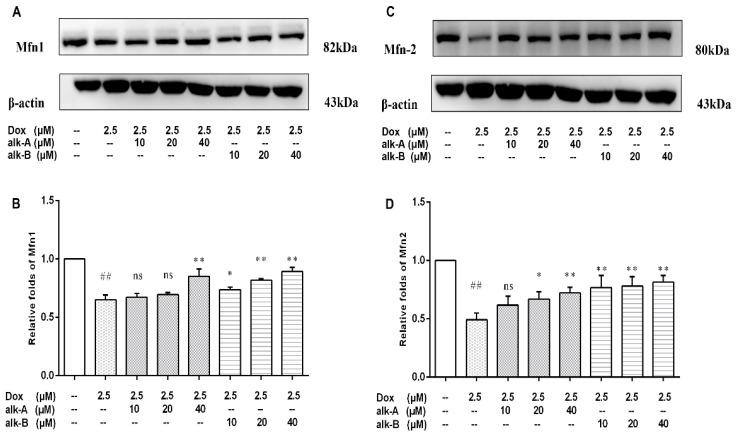
The effects of alkaloids on mitochondrial fusion proteins Mfn-1 (**A**) and Mfn-2 (**B**) during Dox-induced cardiotoxicity. The cells were pretreated with different concentrations of alk-A and alk-B (10, 20, and 40 µM) for 1 h, then exposed to Dox for 24 h. Relative folds of Mfn1 (**C**) and Mfn2 (**D**). Results are mean ± SEM from three independent experiments. ^##^ *p* < 0.01 vs. control, * *p* < 0.05, ** *p* < 0.01 vs. Dox group, ns—no significant difference.

To confirm the results of the mitochondrial biogenesis assessment, immunoblot analysis for key mitochondrial fusion proteins (Mfn1 and Mfn2) were conducted. Dox treatment significantly decreased the protein level of Mfn1 (0.65 ± 0.02-fold, *p* < 0.01) in comparison to the control group as shown in Figure 7. Pre-treatment with 40 μM alk-A followed by Dox treatment resulted in a significant increase in Mfn1 protein level (0.85 ± 0.04-fold vs. 0.65 ± 0.02, *p* < 0.01). Similarly, Mfn2 protein level significantly decreased in response to Dox treated cells in comparison to the control group (0.49 ± 0.03-fold, *p* < 0.01), whereas 20 and 40 μM alk-A pre-treatment significantly increased the protein level of Mfn2 in comparison to the Dox group (0.67 ± 0.04 vs. 0.49 ± 0.03, *p* < 0.05 and 0.72± 0.03 vs. 0.49 ± 0.03, *p* < 0.01). Interestingly, significant changes in the Mfn1 and Mfn2 protein expression compared with Dox group were both observed among the all alk-B treatment groups in a dose-dependent manner. 

#### 2.5.3. Alkaloids Reduce mtDNA Damage in Dox-Treated H9c2 Cells

**Figure 8:**
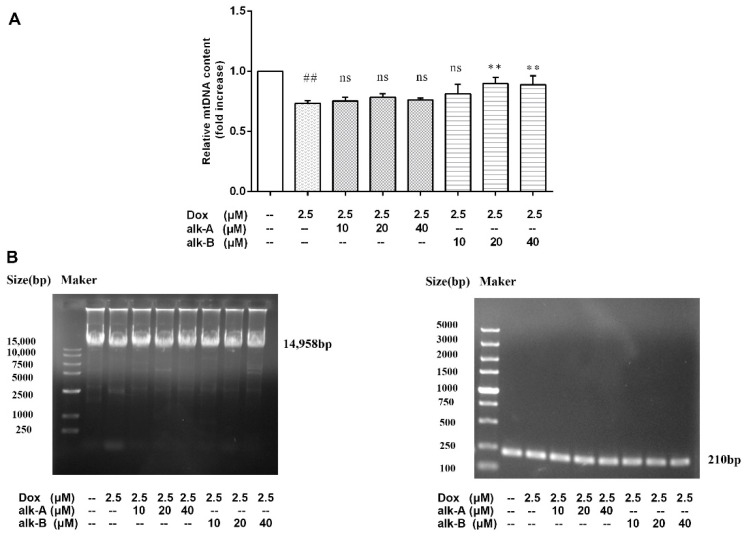
Effects of alkaloids on mtDNA damage in Dox-treated H9c2 cells. mtDNA damage was measured by the ratio of long to short fragments using long PCR (**A**). Cellular mtDNA copy number was assessed by LongPCR analysis (**B**). The cells were pretreated with different concentrations of alk-A and alk-B (10, 20, and 40 µM) for 1 h, then exposed to Dox for 24 h. Results are mean ± SEM from three independent experiments. ^##^ *p* < 0.01 vs. control, * *p* < 0.05, ** *p* < 0.01 vs. Dox group, ns—no significant difference.

The use of DNA polymerase amplification of long mtDNA products (LRPCR) to study mtDNA damage was adapted by our groups. The copy number derived from this amplification was compared to the copy number of a short PCR product (150–250 bp) that was unlikely to contain any lesions. The mtDNA lesion frequency was then calculated from the relative ratio of long:short amplicons. Figure 8 showed that mtDNA damage occurred in Dox-treated H9c2 cells, and a significantly different about mtDNA copy number was observed between Dox-treated and control groups (0.73 ± 0.01-fold vs. control group, *p* < 0.01). As shown in Figure 8, treatment with alk-A (10, 20, and 40 μM) increased the number of mtDNA copies, but showed no significantly different effects on the mtDNA copy number under the same conditions compared to Dox-treated group (0.75 ± 0.02, 0.78 ± 0.02, and 0.76 ± 0.01 vs. 0.73 ± 0.01, *p* > 0.05). Like alk-A, alk-B (20 and 40 μM) increased the number of mtDNA copies and displayed significantly different effects on the mtDNA copy number compared to Dox-treated group (0.89 ± 0.03, 0.89 ± 0.04, and 0.76 ± 0.01 vs. 0.73 ± 0.01, *p <* 0.01). Figure 8 shows that both alk-A and alk-B may protect against mitochondrial DNA damage in different concentrations, with 40 μM alk-B group working best.

## 3. Discussion

Although doxorubicin is an effective anticancer chemotherapeutic agent, its clinical use is limited by cumulative dose-related cardiotoxicity, which may lead to severe and irreversible cardiomyopathy and heart failure [18,25,26]. It is widely accepted that doxorubicin generates great free radicals that are critical inducers of cell death [27]. In this study, we aimed to assess the effect of alkaloids from seabuckthorn seed residue pretreatment on Dox-induced apoptosis in H9c2 cells. The results showed that alkaloids themselves were nontoxic in the range of the trial concentration. Alkaloids have been described as an inhibitor of Dox-induced H9c2 cell apoptosis in several reports [19,28], so it may serve as a potential protective agent against the Dox-mediated apoptosis in H9c2 cardiac myoblast cells. Alk-A and alk-B both are new natural compounds that were first discovered by our research group. Thus, if they can protect against the cardiotoxicity induced by Dox still remains unclear.

In this study, we established a Dox-induced cardiomyocyte injury model, made by exposing H9c2 cells to Dox at 2.5 µM that was within the clinically accepted range of 0.1 to 5 µM in vitro and chosen for further experiments [29]. The results manifested as decreased cell viability, and increased apoptosis, p-JNK expression, ROS production and mitochondrial dysfunction. We first assessed the effects of 10, 20, and 40 µM alk-A and alk-B on Dox-induced cardiotoxicity after 24 h of treatment, respectively. To confirm the potential protective effect of alk-A and alk-B, as noted in MTT assays, the results indicate that alk-A and alk-B both significantly protected H9c2 cells from doxorubicin-induced cytotoxicity. The semiquinone form of Dox is a toxic short-lived metabolite, which in turn can react with O_2_-producing ROS [30]. ROS are proposed to be responsible for Dox-induced apoptosis in cardiac cells [31]. The activation of caspase-3 is a vital step in Dox-induced apoptosis [32]. We then explored the protective effect of alk-A and alk-B against cardiomyocyte apoptosis. The results showed that the protective effect was mediated through the de-creasement of intracellular ROS levels (Figure 3) and the significant inhibition of apopto-sis-related cleaved-caspase-3 protein expression level in 2.5 μM Dox-induced H9c2 cells in a dose-dependent manner (Figure 4).

Mitochondria are abundant in cardiac tissue, constituting about 45% of the myocardial volume in comparison with other tissues [33]. One report has shown that the mitochondrial electron transport chain is the major source for ROS generation in cardiac cells [34]. The heart is particularly susceptible to oxidative stress because of its high energy demand that was satisfied by mitochondrial respiration which generates ATP. ROS generation in response to DOX led to significant dissipation of ATP in the present study. Treatment with alk-A and alk-B significantly ameliorated this phenomenon, especially alk-B in a dose-dependent manner (Figure 6). Generally, the mtDNA copy number per mitochondrion is considered to be constant in all mammalian cell types Therefore, it represents the cellular mitochondrial number [35]. However, mtDNA is more susceptible to oxidative damage than nDNA due to its incomplete DNA repair capacity and the proximity of mtDNA to the respiratory chain, which leads to mitochondrial dysfunction and the activation of the extrinsic apoptotic pathway [9,36,37]. We studied mtDNA damage using long PCR, as shown in Figure 8. mtDNA was damaged in Dox-induced cardiomyopathy, which would result in impaired expression of respiratory chain complex proteins that further led to more ROS generation. Then, a vicious circle was formed. This bad state could be changed through treatment with alk-A and alk-B, with alk-B showing the best effect. Dox-induced mitochondrial fragmentation is associated with the upregulation of dynamin-related protein 1 (Drp1) and downregulation of fusion proteins (Mfn1 and Mfn2), which leads to *cytochrome c* release and activation of caspases [38,39]. Key mitochondrial fusion proteins (Mfn1 and Mfn2) were conducted to confirm the mitochondrial network assessment. In in vitro models, pre-treatment with alk-A and alk-B dose-dependently increased the expression of Mfn1, Mfn2 that were inhibited by Dox (Figure 7). The results shown that alkaloids elicit a protective effect on the mitochondria during stressful conditions such as Dox-induced cardiotoxicity.

Although some antioxidants can reduce Dox-induced enhancement of ROS levels, addition of various antioxidants, including vitamin C, superoxide dismutase, N-acetylcysteine, glutathione, catalase, and probucol did not diminish the cytotoxicity of Dox in a variety of tumor cells [32]. However, our previous in vitro studies showed that the antioxidant activity of these two alkaloids was not excellent, so we speculated that this protective effect in Dox-induced cardiotoxicity may be achieve through other cellular signaling pathways. ROS acts as a stimulus and modulates mitogenic-activated protein kinase (MAPK) pathways which comprise extracellular-regulated (ERKs), c-jun-NH2-terminal kinase (JNKs), and p38 MAPK [40]. Results of several studies indicate that JNK activation can be proapoptotic in Dox-treated cardiac muscle cells [41,42,43]. In our study, alk-A- and alk-B-treated H9c2 cells significantly inhibited the Dox-induced activation JNK, which was in correspondence with previous experimental results. A study has shown that alk-B showed potent anti-inflammatory activities, with IC_50_ values of 25.16 ± 0.41 µM on LPS (lipopolysaccharide)-induced RAW264.7 cells [44]. As well, alk-A exhibited significant anti-neuroinflammatory activity on TNF-α release from LPS-induced BV2 microglia cells [45]. Therefore, the inhibition effect of phosphorylation JNK1/2/3 of alk-A and alk-B may be attributed to anti-inflammatory activity via regulating signaling cascades of NF-κB. That is why we are going to focus on the phosphorylation of JNK to elucidate the mechanism of Dox-induced apoptosis.

## 4. Materials and Methods

### 4.1. Materials

The H9c2 cell line was purchased from the cell bank of the Institute of Biochemistry and Cell Biology of Shanghai (Shanghai, China) and grown in Dulbecco’s modified Eagle’s medium (DMEM; Gibco, Carlsbad, CA, USA) containing 10% fetal bovine serum (FBS; Gibco, Carlsbad, CA, USA) and 1% penicillin streptomycin (Gibco, Grand Island, NY, USA). 3-(4,5-dimethylthiazol-2yl)-2,5 diphenyltetrazoliumbromide (MTT) was bought from Sigma Aldrich Inc. (St Louis, MO, USA). Doxorubicin and luminescence-enhanced ATP assay kit were purchased from Beyotime Biotech Inc. (Shanghai, China). 2′,7′-Dichlorofluorescein diacetate (DCFH-DA) was obtained from Thermo Fisher Scientific Inc. (Rockford, IL, USA). DNA extraction kit (TIANGEN, Beijing, China), Long Amp Taq 2× Master Mix (NEB, Ipswitch, MA, USA) and Taq DNA Polymerase (Thermo Fisher Scientific, Waltham, MA, USA) were used for mitochondrial DNA damage assay. Primary antibodies for p-JNK, JNK, Mfn1, and Mfn2 were purchased from Abcam Inc. (Cambridge, MA, USA). Primary antibodies for β-actin and caspase-3 were purchased from Cell Signaling Technology (Beverly, MA, USA), HRP goat anti-rabbit antibody and HRP goat anti-mouse antibody were purchased from Beijing BioDee Biotechnology Co., Ltd. (Beijing, China).

### 4.2. Preparation of Purifed Compounds

The isolation and purification of alkaloids from seed residue of *Hippophae rhamnoides* L. was carried out as described by Ouyang et al. [24]. Chemical structures of alkaloids from *Hippophae rhamnoides* L. are shown in Figure 1. The isolated alkaloids (alk-A and alk-B) were prepared in DMSO(dimethyl sulphoxide) and stored at −20 °C.

### 4.3. Cell Culture

H9c2 embryonic rat cardiac cells (H9c2 cells) were cultured in DMEM supplemented with 10% FBS, 100 U/mL of penicillin, 100 μg/mL of streptomycin, and 5% CO_2_ at 37 °C. The cells were fed every 2–3 days and subcultured once they reached 80% confluence. Cells were plated at an appropriate density according to each experimental design. For the experiments, they were seeded in 96-, 24-, and 6-well culture plates for various assays, respectively. To explore the protective effects of alkaloids on the Dox-induced injury, the cells were pretreated with alk-A and alk-B (10, 20, and 40 µM) for 1 h and then incubation was continued in the presence of the alkaloids with 2.5 µM doxorubicin for 24 h.

### 4.4. Cell Viability Assay

Initially, a pilot study was performed to determine a concentration of Dox that would induce cardiotoxicity. Based on the above results, a dose-response study was performed to determine a concentration of alkaloids that would reduce the toxicity in H9c2 cells treated with doxorubicin (2.5 µM). Alkaloids were added to H9c2 cell cultures at the desired concentration 1 h before treatment with doxorubicin and then incubation was continued with 2.5 µM doxorubicin for 24 h. The cell viability was determined using a modified MTT assay as described previously. The MTT solution (5 mg/mL) was added to each well and the cells were incubated for 3 h in the incubator. The resultant formazan product was dissolved by the addition of 100 μL of DMSO. Absorbance was detected at 570 nm using a Multi-Mode Detection Platform (Molecular Devices, San Jose, CA, USA). The experiment was carried out in triplicate.

### 4.5. Determination of Intracellular ROS Generation

DCFH-DA was used to determine the production of intracellular reactive oxygen species (ROS) with flow cytometry. After treatment, cells were incubated with 10 μM DCFH-DA in the dark under 37 °C for 30 min, washed twice with pre-warmed PBS, and then analyzed using a NovoCyte 2040R flow cytometer (San Diego, CA, USA), with excitation/emission wavelengths of 488 nm excitation and 525 nm emission wavelengths. The experiment was performed in triplicate.

### 4.6. Western Blot Analysis

For Western blot experiments, cells were washed twice with ice-cold PBS to remove the excess of culture medium, then were lysed for 30 min in ice-cold RIPA buffer (50 mM Tris-HCl (pH 7.4), 150 mM NaCl, 1% NP-40, 0.1% SDS, 1 mM phenylmethyl sulphonyl fluoride (PMSF)), cells were harvested by scraping. Lysates were centrifuged at 12,000× *g* for 20 min at 4 °C, and supernatants were collected for analysis. The protein concentration was measured in the supernatant using the Pierce-23225 BCA Protein Assay Kit. The proteins (30 μg) were separated via 10–15% SDS-PAGE gel electrophoresis and transferred onto a PVDF (Polyvinylidene Fluoride) membrane (Millipore, Bedford, MA, USA) at 250 mA for 1 h using a Bio-RAD electrophoresis equipment (Miniprotean; Bio-Rad, Hercules, CA, USA). The blots were blocked with 5% skim milk in TBS (Tris-buffered saline) (10 mM Tris-base, pH 7.5, 68 mM NaCl) containing 0.05% Tween 20 for 1 h at room temperature, after washing for 10 min three times with TBST, then hybridized with primary antibodies (diluted 1:1000 in the blocking solution) overnight at 4 °C. Subsequently, the blots were incubated with peroxidase-conjugated antibodies for 1 h at room temperature. Western blot bands were visualized using a 5200 Multi Luminescent Image Analyzer (Tanon Science & Technology Co., Ltd., Shanghai, China).

### 4.7. ATP Content Measurement

For ATP determination, wells containing doxorubicin should be washed and refilled with ordinary media just prior to measurement [46]. ATP content was measured using a luciferase-based luminescence-enhanced ATP assay kit. According to the manufacturer’s instructions, cells were washed twice with ice-cold PBS and homogenized in an ice-cold ATP releasing buffer. ATP concentrations were then determined using an ATP standard with a SpectraMax Paradigm Multi-Mode Microplate Reader (Molecular Devices Corporation, Sunnyvale, CA, USA).

### 4.8. Detection of mtDNA Damage by Long PCR

DNA was isolated from the H9c2 cell using a DNA extraction kit and then quantified by spectrophotometry at 260 nm and qualified by 0.7% agarose gel electrophoresis, respectively. Long PCR experiments allowed the detection of mtDNA lesions that hamper the progression of polymerases and altering replication [47,48]. The long PCR technique is based on the amplification of a long (14,958-bp) and a short (210-bp) mtDNA fragments. Primer sequences were as follows: LRPCR: forward, 5′-ATTTTCTCCCAGTTACGAAAG-3′; reverse, 5′-CTTGGTAAGTAAATTTCTTTCTCC-3′; SRPCR: forward, 5′-ATGCACGATAGCTAAGACCCAA-3′; reverse, 5′-GCTGAATTAGCGAGAAGGGGTA-3′. PCR reactions were performed with the Veriti 96-well thermal cycler long PCR system (Applied Biosystems, Foster City, CA), as recommended by the manufacturer. The final LRPCR cycling parameters were initial denaturation at 94 °C for 10 min followed by 45 s at 94 °C, 10 s at 61 °C, 8 min at 68 °C for 40 cycles, and a final extension at 72 °C for 10 min using Long Amp Taq 2× Master Mix (NEB, Ipswitch, Burlington, MA, USA). The short fragment reaction thermocycler profile included initial denaturation at 94 °C for 10 min, 30 cycles of 94 °C for 45 s, 56 °C for 45 s, 72 °C for 45 s, and a final extension at 72 °C for 10 min using Taq DNA Polymerase (Thermo Fisher Scientific, Massachusetts, USA). PCR products were subjected to electrophoresis on ethidium-bromide-containing agarose gels, and the intensity of each PCR fragment was determined using a transilluminator (Tanon Science & Technology Co., Ltd., Shanghai, China). The long/short mtDNA intensity ratio was thus calculated for each sample.

### 4.9. Statistics

All data were expressed as mean ± SEM from three independent experiments. Statistical analysis was performed using one way analysis of variance (ANOVA) or Student’s *t* test using the statistical analysis software SPSS18.0. Significant changes are denoted as follows: * *p* < 0.05; ** *p* < 0.01.

## 5. Conclusions

In conclusion, our results demonstrated that alk-A and alk-B both exhibited a protective effect against Dox-induced cytotoxicity in H9c2 cardiomyocytes, while alk-B showed more protection. Our results showed that alkaloids from seabuckthorn seed residue can inhibit cell apoptosis and improve mitochondrial function in Dox-induced cardiotoxicity, by suppressing cleaved-caspase-3 protein expression, intracellular reactive oxygen species (ROS) production, and activation of JNK, and increasing ATP levels, favoring mitochondrial mitofusin protein expression and relieving damage to mitochondrial DNA. Therefore, our observations suggest that alk-B may be an attractive candidate as a Dox-induced cytotoxicity-improving agent for clinical treatment. However, more investigations are needed to elucidate the probable underlying mechanisms of these valuable effects.

## Data Availability

The data presented in this study are available on request from the corresponding author.

## References

[1] Ragavendran P., Sophia D., Arulraj C., Gopalakrishnan V.K. (2012). Cardioprotective effect of aqueous, ethanol and aqueous ethanol extract of *Aerva lanata* (Linn.) against doxorubicin induced cardiomyopathy in rats. Asian Pac. J. Trop. Biomed..

[2] Wu X., Mao Y. (2012). Effect of catalpol on doxorubicin-induced cytotoxicity in H9c2 cells. J. Med. Plants Res..

[3] Swain S.M., Whaley F.S., Ewer M.S. (2010). Congestive heart failure in patients treated with doxorubicin: A retrospective analysis of three trials. Cancer.

[4] Govender Y.J., Loos B., Marais E., Engelbrecht A.M. (2014). Mitochondrial catastrophe during doxorubicin-induced cardiotoxicity: A review of the protective role of melatonin. J. Pineal Res..

[5] Minotti G., Menna P., Salvatorelli E., Cairo G., Gianni L. (2004). Anthracyclines: Molecular advances and pharmacologic developments in antitumor activity and cardiotoxicity. Pharmacol. Rev..

[6] Olson R.D., Mushlin P.S. (1990). Doxorubicin cardiotoxicity: Analysis of prevailing hypotheses. FASEB J..

[7] Singal P., Iliskovic N., Li T., Kumar D. (1997). Adriamycin cardiomyopathy: Pathophysiology and prevention. FASEB J..

[8] Kalyanaraman B., Joseph J., Kalivendi S., Wang S., Konorev E., Kotamraju S. (2002). Doxorubicin-induced apoptosis: Implications in cardiotoxicity. Mol. Cell. Biochem..

[9] Tokarska-Schlattner M., Zaugg M., Zuppinger C., Wallimann T., Schlattner U. (2006). New insights into doxorubicin-induced cardiotoxicity: The critical role of cellular energetics. J. Mol. Cell. Cardiol..

[10] Green D.R., Reed J.C. (1998). Mitochondria and apoptosis. Science.

[11] Sainz R., Mayo J., Rodriguez C., Tan D. (2003). Lopez-Burillo, S.; Reiter, R.J. Melatonin and cell death: Differential actions on apoptosis in normal and cancer cells. Cell. Mol. Life Sci..

[12] Sun B., Sun G.B., Xiao J., Chen R.C., Wang X., Wu Y., Cao L., Yang Z.H., Sun X.B. (2012). Isorhamnetin inhibits H_2_O_2_-induced activation of the intrinsic apoptotic pathway in H9c2 cardiomyocytes through scavenging reactive oxygen species and ERK inactivation. J. Cell. Biochem..

[13] Zhou W., Yuan Z., Li G., Ouyang J., Suo Y., Wang H. (2018). Isolation and structure determination of a new flavone glycoside from seed residues of seabuckthorn (*Hippophae rhamnoides* L.). Nat. Prod. Res..

[14] Hu C.Y., Xu D.P. (2010). Extraction, Isolation and Protective Effect of Alkaloid from Seabuckthorn Seeds on Injured Cardiomyocytes in Rats. Food Sci..

[15] Sun J., Sun G., Meng X., Wang H., Luo Y., Qin M., Ma B., Wang M., Cai D., Guo P. (2013). Isorhamnetin protects against doxorubicin-induced cardiotoxicity in vivo and in vitro. PLoS ONE.

[16] Koneru M., Nalban N., Sahu B.D., Sistla R. (2017). Natural Products against Drug-Induced Cardiotoxicity. Promises and Hopes.

[17] Gulimire A., Ybadaiti T., Rena K. (2010). Protective effect of total flavonoids of H. rhamnoides L. subsp. turkestanica Rousi against adriamycin-induced cardiotoxicity in rats. J. Xinjiang Med. Univ..

[18] Carvalho F.S., Burgeiro A., Garcia R., Moreno A.J., Carvalho R.A., Oliveira P.J. (2014). Doxorubicin-induced cardiotoxicity: From bioenergetic failure and cell death to cardiomyopathy. Med. Res. Rev..

[19] Priya L.B., Baskaran R., Huang C.Y., Padma V.V. (2017). Neferine ameliorates cardiomyoblast apoptosis induced by doxorubicin: Possible role in modulating NADPH oxidase/ROS-mediated NFκB redox signaling cascade. Sci. Rep..

[20] Lv X., Yu X., Wang Y., Wang F., Li H., Wang Y., Lu D., Qi R., Wang H. (2012). Berberine inhibits doxorubicin-triggered cardiomyocyte apoptosis via attenuating mitochondrial dysfunction and increasing Bcl-2 expression. PLoS ONE.

[21] Hao G., Yu Y., Gu B., Xing Y., Xue M. (2015). Protective effects of berberine against doxorubicin-induced cardiotoxicity in rats by inhibiting metabolism of doxorubicin. Xenobiotica.

[22] Xin H., Liu X.H., Zhu Y.Z. (2009). Herba leonurine attenuates doxorubicin-induced apoptosis in H9c2 cardiac muscle cells. Eur. J. Pharmacol..

[23] Chatterjee K., Zhang J., Tao R., Honbo N., Karliner J.S. (2008). Vincristine attenuates doxorubicin cardiotoxicity. Biochemical and biophysical research communications. Biochem. Biophys. Res. Commun..

[24] OuYang J., Zhou W.N., Li G., Wang X.Y., Ding C.X., Suo Y.R., Wang H.L. (2015). Three new alkaloids from *Hippophae rhamnoides* Linn. subsp. sinensis Rousi. Helv. Chim. Acta..

[25] Zhang S., Liu X., Bawa-Khalfe T., Lu L.-S., Lyu Y.L., Liu L.F., Yeh E.T. (2012). Identification of the molecular basis of doxorubicin-induced cardiotoxicity. Nat. Med..

[26] Sardão V.A., Oliveira P.J., Holy J., Oliveira C.R., Wallace K.B. (2009). Morphological alterations induced by doxorubicin on H9c2 myoblasts: Nuclear, mitochondrial, and cytoskeletal targets. Cell Biol. Toxicol..

[27] Octavia Y., Tocchetti C.G., Gabrielson K.L., Janssens S., Crijns H.J., Moens A.L. (2012). Doxorubicin-induced cardiomyopathy: From molecular mechanisms to therapeutic strategies. J. Mol. Cell. Cardiol..

[28] Kim D.S., Woo E.R., Chae S.W., Ha K.C., Lee G.H., Hong S.T., Kwon D.Y., Kim M.S., Jung Y.K., Kim H.M. (2007). Plantainoside D protects adriamycin-induced apoptosis in H9c2 cardiac muscle cells via the inhibition of ROS generation and NF-κB activation. Life Sci..

[29] Liu J., Mao W., Bo D., Liang C.S. (2008). ERKs/p53 signal transduction pathway is involved in doxorubicin-induced apoptosis in H9c2 cells and cardiomyocytes. Am. J. Physiol. Heart Circ. Physiol..

[30] Doroshow J.H. (1983). Anthracycline antibiotic-stimulated superoxide, hydrogen peroxide, and hydroxyl radical production by NADH dehydrogenase. Cancer Res..

[31] Mizutani H., Tada-Oikawa S., Hiraku Y., Kojima M., Kawanishi S. (2005). Mechanism of apoptosis induced by doxorubicin through the generation of hydrogen peroxide. Life Sci..

[32] Takemura G., Fujiwara H. (2007). Doxorubicin-induced cardiomyopathy from the cardiotoxic mechanisms to management. Pro. Cardiovasc. Dis..

[33] Marin-Garcia J., Goldenthal M.J., Moe G.W. (2001). Mitochondrial pathology in cardiac failure. Cardiovasc. Res..

[34] Dikalov S.I., Ungvari Z. (2013). Role of mitochondrial oxidative stress in hypertension. Am. J. Physiol Heart Circ Physiol..

[35] Montier L.L.C., Deng J.J., Bai Y. (2009). Number matters: Control of mammalian mitochondrial DNA copy number. Journal of genetics and genomics. J. Genet. Genom..

[36] Berthiaume J.M., Wallace K.B. (2007). Adriamycin-induced oxidative mitochondrial cardiotoxicity. Cell Biol. Toxicol..

[37] Görlach A., Dimova E.Y., Petry A., Martínez-Ruiz A., Pablo H.A., Rolo A.P., Palmeira C.M., Kietzmann T. (2015). Reactive oxygen species, nutrition, hypoxia and diseases: Problems solved?. Redox Biol..

[38] Karbowski M., Youle R.J. (2003). Dynamics of mitochondrial morphology in healthy cells and during apoptosis. Cell Death Differ..

[39] Gharanei M., Hussain A., Janneh O., Maddock H. (2013). Attenuation of Doxorubicin-Induced Cardiotoxicity by mdivi-1: A Mitochondrial Division/Mitophagy Inhibitor. PLoS ONE.

[40] Chen M.B., Wu X.Y., Gu J.H., Guo Q.T., Shen W.X., Lu P.H. (2011). Activation of AMP-Activated Protein Kinase Contributes to Doxorubicin-Induced Cell Death and Apoptosis in Cultured Myocardial H9c2 Cells. Cell Biochem. Biophys..

[41] Kim D.S., Kim H.R., Woo E.R., Hong S.T., Chae H.J., Chae S.W. (2005). Inhibitory effects of rosmarinic acid on adriamycin-induced apoptosis in H9c2 cardiac muscle cells by inhibiting reactive oxygen species and the activations of c-Jun N-terminal kinase and extracellular signal-regulated kinase. Biochem. Pharmacol..

[42] Panaretakis T., Laane E., Pokrovskaja K., Björklund A.C., Dan G. (2005). Doxorubicin Requires the Sequential Activation of Caspase-2, Protein Kinase Cdelta, and c-Jun NH2-terminal Kinase to Induce Apoptosis. Mol. Biol. Cell..

[43] Brantley-Finley C., Lyle C.S., Du L., Goodwin M.E., Hall T., Szwedo D., Kaushal G.P., Chambers T.C. (2003). The JNK, ERK and p53 pathways play distinct roles in apoptosis mediated by the antitumor agents vinblastine, doxorubicin, and etoposide. Biochem. Pharmacol..

[44] Wang C., Hyun-Jae J., Yoo H., Xiang S., Seung L., Mun-Chual R., Heng-Shan W., Seo Y., Young K. (2018). Alkaloids from Tetrastigma hemsleyanum and Their Anti-Inflammatory Effects on LPS-Induced RAW264.7 Cells. Molecules.

[45] Zhou Y., Wang S., Lou H., Fan P. (2018). Chemical constituents of hemp (*Cannabis sativa* L.) seed with potential anti-neuroinflammatory activity. Phytochem. Lett..

[46] Calvert R.J., Vohra S. (2013). Doxorubicin-treated H9c2 cells: Caution with luminescent ATP and Hoechst 33258 assays. In Vitro Cell Dev. Biol. Anim..

[47] Santos J.H., Mandavilli B.S., Van Houten B. (2002). Measuring Oxidative mtDNA Damage and Repair Using Quantitative PCR. Methods Mol. Biol..

[48] Larosche I., Lettéron P., Berson A., Fromenty B., Huang T.-T., Moreau R., Pessayre D., Mansouri A. (2010). Hepatic mitochondrial DNA depletion after an alcohol binge in mice: Probable role of peroxynitrite and modulation by manganese superoxide dismutase. J. Pharmacol. Exp. Ther..

